# *N*-[(3*RS*,4*SR*)-1-Benzyl-4-methyl­piperi­din-3-yl]-1-(4-methylphenyl­sulfonyl)-5-nitro-1*H*-pyrrolo­[2,3-*b*]pyridin-4-amine

**DOI:** 10.1107/S160053681203961X

**Published:** 2012-09-26

**Authors:** Ellen Pfaffenrot, Dieter Schollmeyer, Stefan Laufer

**Affiliations:** a Eberhard-Karls-University Tübingen, Auf der Morgenstelle 8, 72076 Tübingen, Germany; b University Mainz, Institute of Organic Chemistry, Duesbergweg 10-14, 55099 Mainz, Germany

## Abstract

The structure of the title compound, C_27_H_29_N_5_O_4_S, displays an intra­molecular N—H⋯O hydrogen bond. The pyrrolo­[2,3-*b*]pyridine core makes a dihedral angle of 85.5 (4)° with the benzyl residue and a dihedral angle of 89.4 (9)° with the tosyl ring. The nitro group is slightly twisted out of the plane of the planar pyrrolo­pyridine system [(—N—)C—C—N—O torsion angle = −4.61 (18)° and (—NH—)C—C—N—O = −6.46 (18)°].

## Related literature

For inhibitors of Janus kinases, see: Hoffmann-La Roche (2011 ▶).
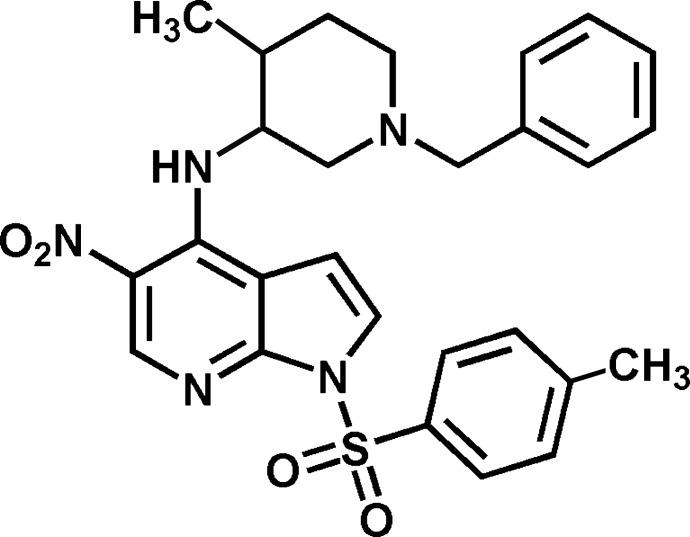


## Experimental

### 

#### Crystal data

C_27_H_29_N_5_O_4_S
*M* *_r_* = 519.61
Monoclinic, 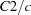

*a* = 29.5164 (12) Å
*b* = 9.1388 (2) Å
*c* = 23.3008 (9) Å
β = 124.803 (3)°
*V* = 5160.9 (3) Å^3^
*Z* = 8
Mo *K*α radiation
μ = 0.17 mm^−1^
*T* = 193 K
0.55 × 0.32 × 0.30 mm

#### Data collection

Stoe IPDS 2T diffractometer
32401 measured reflections
6211 independent reflections
5273 reflections with *I* > 2σ(*I*)
*R*_int_ = 0.049

#### Refinement

*R*[*F* ^2^ > 2σ(*F* ^2^)] = 0.035
*wR*(*F* ^2^) = 0.101
*S* = 1.03
6211 reflections
336 parameters
H-atom parameters constrained
Δρ_max_ = 0.32 e Å^−3^
Δρ_min_ = −0.40 e Å^−3^

### 

Data collection: *X-AREA* (Stoe & Cie, 2010 ▶); cell refinement: *X-AREA*; data reduction: *X-RED* (Stoe & Cie, 2010 ▶); program(s) used to solve structure: *SIR97* (Altomare *et al.*, 1999 ▶); program(s) used to refine structure: *SHELXL97* (Sheldrick, 2008 ▶); molecular graphics: *PLATON* (Spek, 2009 ▶); software used to prepare material for publication: *PLATON*.

## Supplementary Material

Crystal structure: contains datablock(s) I, global. DOI: 10.1107/S160053681203961X/bt6839sup1.cif

Structure factors: contains datablock(s) I. DOI: 10.1107/S160053681203961X/bt6839Isup2.hkl

Supplementary material file. DOI: 10.1107/S160053681203961X/bt6839Isup3.cml

Additional supplementary materials:  crystallographic information; 3D view; checkCIF report

## Figures and Tables

**Table 1:** Hydrogen-bond geometry (Å, °)

*D*—H⋯*A*	*D*—H	H⋯*A*	*D*⋯*A*	*D*—H⋯*A*
N23—H23⋯O21	0.87	1.93	2.6274 (14)	137

**Fig. 1.**
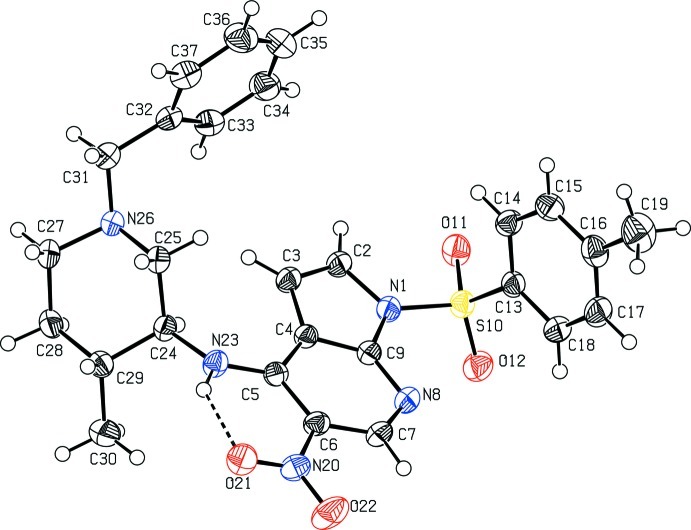
Crystal structure of the title compound with labelling and displacement ellipsoids drawn at the 50% probability level. Hydrogen bond is represented as dashed lines.

## supplementary crystallographic information

### Comment

*N*-(1-Benzyl-4-methylpiperidin-3-yl)-5-nitro-1-tosyl-1*H*-pyrrolo[2,3-*b*]pyridine-4-amine is an important intermediate in the synthesis of tricyclic heterocyclic compounds being inhibitors of Janus kinases (Hoffmann-La Roche, 2011).

The pyrrolo-pyridine system shows a dihedral angle of 85.5 (4)° towards the benzyl residue and 89.4 (9)° towards the tosyl ring. The methylene group in the title compound presents an angle of 74.4 (9)° between the phenyl and piperidine residue. The equatorial substituents of the piperidine are in *trans* configuration displaying a torsion angle of 57.4 (2)° (Fig. 1). The intramolecular hydrogen bond N23—H23···O21 1.92 Å stabilize the molecular conformation (Table 1).

### Experimental

The compound was prepared by nucleophilic substitution of 4-chloro-5-nitro-1-tosyl-1*H*-pyrrolo[2,3-*b*]pyridine with *trans*-1-benzyl-3-aminopiperidine in the presence of tetriary amine base. A mixture of 4-chloro-5-nitro-1-tosyl-1*H*-pyrrolo[2,3-*b*]pyridine (0.213 g, 0.607 mmol), *trans*-1-benzyl-3-aminopiperidine-hydrochloride (0.335 g, 1.214 mmol) and diisopropylethylamine (0.6 ml, 3.641 mmol) in dioxane (2 ml) was heated in a microwave reactor at 373 K for 1 h. The reaction media was concentrated under vacuo and purrified by column chromatography (SiO_2_, n-hexane / ethyl acetate; 3:1).

### Refinement

All H atoms were visible in a difference map. Hydrogen atoms attached to carbons were placed at calculated positions with C—H = 0.95 Å (aromatic) or 0.98–0.99 Å (*sp*^3^ C-atom). All H atoms were refined in the riding-model approximation with isotropic displacement parameters set at 1.2–1.5 times of the *U*_eq_ of the parent atom.

### Crystal data

**Table:**

C_27_H_29_N_5_O_4_S	*F*(000) = 2192
*M**_r_* = 519.61	*D*_x_ = 1.337 Mg m^−^^3^
Monoclinic, *C*2/*c*	Mo *K*α radiation, λ = 0.71073 Å
Hall symbol: -C 2yc	Cell parameters from 28567 reflections
*a* = 29.5164 (12) Å	θ = 3.1–29.5°
*b* = 9.1388 (2) Å	µ = 0.17 mm^−^^1^
*c* = 23.3008 (9) Å	*T* = 193 K
β = 124.803 (3)°	Block, yellow
*V* = 5160.9 (3) Å^3^	0.55 × 0.32 × 0.30 mm
*Z* = 8	

### Data collection

**Table:**

Stoe IPDS 2T diffractometer	5273 reflections with *I* > 2σ(*I*)
Radiation source: sealed Tube	*R*_int_ = 0.049
Graphite monochromator	θ_max_ = 28.0°, θ_min_ = 3.1°
Detector resolution: 6.67 pixels mm^-1^	*h* = −38→38
rotation method scans	*k* = −12→10
32401 measured reflections	*l* = −30→30
6211 independent reflections	

### Refinement

**Table:**

Refinement on *F*^2^	Primary atom site location: structure-invariant direct methods
Least-squares matrix: full	Secondary atom site location: difference Fourier map
*R*[*F*^2^ > 2σ(*F*^2^)] = 0.035	Hydrogen site location: inferred from neighbouring sites
*wR*(*F*^2^) = 0.101	H-atom parameters constrained
*S* = 1.03	*w* = 1/[σ^2^(*F*_o_^2^) + (0.0532*P*)^2^ + 2.4419*P*] where *P* = (*F*_o_^2^ + 2*F*_c_^2^)/3
6211 reflections	(Δ/σ)_max_ = 0.002
336 parameters	Δρ_max_ = 0.32 e Å^−^^3^
0 restraints	Δρ_min_ = −0.40 e Å^−^^3^

### Special details

**Table:**

Geometry. All e.s.d.'s (except the e.s.d. in the dihedral angle between two l.s. planes) are estimated using the full covariance matrix. The cell e.s.d.'s are taken into account individually in the estimation of e.s.d.'s in distances, angles and torsion angles; correlations between e.s.d.'s in cell parameters are only used when they are defined by crystal symmetry. An approximate (isotropic) treatment of cell e.s.d.'s is used for estimating e.s.d.'s involving l.s. planes.
Refinement. Refinement of *F*^2^ against ALL reflections. The weighted *R*-factor *wR* and goodness of fit *S* are based on *F*^2^, conventional *R*-factors *R* are based on *F*, with *F* set to zero for negative *F*^2^. The threshold expression of *F*^2^ > σ(*F*^2^) is used only for calculating *R*-factors(gt) *etc*. and is not relevant to the choice of reflections for refinement. *R*-factors based on *F*^2^ are statistically about twice as large as those based on *F*, and *R*- factors based on ALL data will be even larger.

### Fractional atomic coordinates and isotropic or equivalent isotropic displacement parameters (Å^2^)

**Table:**

	*x*	*y*	*z*	*U*_iso_*/*U*_eq_	
N1	0.36895 (4)	0.36468 (12)	0.43728 (5)	0.0298 (2)	
C2	0.37391 (5)	0.34971 (14)	0.38137 (6)	0.0310 (2)	
H2	0.4055	0.3137	0.3847	0.037*	
C3	0.32669 (5)	0.39448 (14)	0.32195 (6)	0.0300 (2)	
H3	0.3192	0.3940	0.2765	0.036*	
C4	0.28973 (5)	0.44294 (13)	0.33962 (6)	0.0261 (2)	
C5	0.23605 (5)	0.50601 (12)	0.30208 (6)	0.0263 (2)	
C6	0.21875 (5)	0.54658 (13)	0.34591 (6)	0.0292 (2)	
C7	0.25185 (5)	0.52100 (15)	0.41836 (6)	0.0330 (3)	
H7	0.2375	0.5499	0.4442	0.040*	
N8	0.30113 (4)	0.46032 (12)	0.45339 (5)	0.0330 (2)	
C9	0.31769 (5)	0.42474 (13)	0.41213 (6)	0.0273 (2)	
S10	0.420371 (12)	0.33155 (3)	0.521638 (15)	0.03107 (9)	
O11	0.46345 (4)	0.27462 (11)	0.51761 (5)	0.0388 (2)	
O12	0.39824 (4)	0.24718 (11)	0.55130 (5)	0.0416 (2)	
C13	0.43840 (5)	0.50589 (14)	0.55916 (6)	0.0299 (2)	
C14	0.46497 (5)	0.60144 (14)	0.54113 (6)	0.0323 (3)	
H14	0.4728	0.5728	0.5084	0.039*	
C15	0.47988 (5)	0.73842 (15)	0.57134 (7)	0.0352 (3)	
H15	0.4980	0.8044	0.5592	0.042*	
C16	0.46866 (5)	0.78136 (16)	0.61955 (7)	0.0383 (3)	
C17	0.44216 (6)	0.68377 (17)	0.63658 (7)	0.0414 (3)	
H17	0.4346	0.7120	0.6695	0.050*	
C18	0.42645 (5)	0.54577 (16)	0.60664 (7)	0.0373 (3)	
H18	0.4079	0.4801	0.6183	0.045*	
C19	0.48486 (8)	0.93192 (19)	0.65147 (10)	0.0573 (4)	
H19A	0.4532	0.9789	0.6471	0.086*	
H19B	0.4967	0.9908	0.6271	0.086*	
H19C	0.5153	0.9240	0.7010	0.086*	
N20	0.16570 (5)	0.61010 (12)	0.31848 (6)	0.0345 (2)	
O21	0.13535 (4)	0.64328 (11)	0.25557 (5)	0.0413 (2)	
O22	0.15099 (5)	0.62928 (15)	0.35750 (6)	0.0555 (3)	
N23	0.20496 (4)	0.52638 (12)	0.23263 (5)	0.0304 (2)	
H23	0.1735	0.5677	0.2169	0.036*	
C24	0.21171 (5)	0.45266 (13)	0.18239 (6)	0.0273 (2)	
H24	0.2346	0.3629	0.2050	0.033*	
C25	0.24124 (5)	0.55058 (13)	0.16051 (6)	0.0297 (2)	
H25A	0.2178	0.6361	0.1343	0.036*	
H25B	0.2762	0.5870	0.2025	0.036*	
N26	0.25288 (4)	0.46765 (12)	0.11666 (5)	0.0312 (2)	
C27	0.20055 (5)	0.42158 (17)	0.05248 (6)	0.0379 (3)	
H27A	0.2084	0.3638	0.0232	0.045*	
H27B	0.1793	0.5093	0.0256	0.045*	
C28	0.16618 (5)	0.33024 (16)	0.06856 (7)	0.0360 (3)	
H28A	0.1854	0.2364	0.0896	0.043*	
H28B	0.1304	0.3077	0.0243	0.043*	
C29	0.15545 (5)	0.40571 (14)	0.11824 (6)	0.0319 (2)	
H29	0.1328	0.4953	0.0948	0.038*	
C30	0.12401 (6)	0.30586 (18)	0.13668 (8)	0.0464 (3)	
H30A	0.0884	0.2794	0.0939	0.070*	
H30B	0.1179	0.3571	0.1686	0.070*	
H30C	0.1456	0.2170	0.1593	0.070*	
C31	0.28426 (6)	0.55550 (18)	0.09791 (7)	0.0415 (3)	
H31A	0.2625	0.6438	0.0724	0.050*	
H31B	0.2890	0.4979	0.0657	0.050*	
C32	0.34032 (5)	0.60269 (15)	0.15957 (6)	0.0319 (3)	
C33	0.37477 (6)	0.50706 (15)	0.21396 (7)	0.0359 (3)	
H33	0.3623	0.4109	0.2134	0.043*	
C34	0.42697 (6)	0.54990 (18)	0.26888 (8)	0.0429 (3)	
H34	0.4499	0.4836	0.3059	0.051*	
C35	0.44582 (6)	0.68891 (19)	0.26995 (8)	0.0478 (4)	
H35	0.4816	0.7187	0.3077	0.057*	
C36	0.41230 (6)	0.78400 (17)	0.21587 (8)	0.0460 (3)	
H36	0.4253	0.8793	0.2161	0.055*	
C37	0.35990 (6)	0.74160 (15)	0.16128 (7)	0.0375 (3)	
H37	0.3371	0.8085	0.1245	0.045*	

### Atomic displacement parameters (Å^2^)

**Table:**

	*U*^11^	*U*^22^	*U*^33^	*U*^12^	*U*^13^	*U*^23^
N1	0.0277 (5)	0.0355 (5)	0.0262 (5)	0.0006 (4)	0.0153 (4)	−0.0020 (4)
C2	0.0302 (6)	0.0359 (6)	0.0299 (6)	0.0009 (5)	0.0190 (5)	−0.0035 (5)
C3	0.0314 (6)	0.0334 (6)	0.0287 (5)	0.0011 (5)	0.0192 (5)	−0.0019 (5)
C4	0.0277 (5)	0.0265 (5)	0.0263 (5)	−0.0018 (4)	0.0167 (4)	−0.0022 (4)
C5	0.0287 (5)	0.0242 (5)	0.0281 (5)	−0.0019 (4)	0.0174 (5)	−0.0025 (4)
C6	0.0297 (6)	0.0305 (6)	0.0326 (6)	0.0007 (5)	0.0208 (5)	−0.0018 (5)
C7	0.0363 (6)	0.0389 (7)	0.0323 (6)	−0.0007 (5)	0.0246 (5)	−0.0033 (5)
N8	0.0347 (5)	0.0402 (6)	0.0288 (5)	−0.0006 (4)	0.0207 (4)	−0.0028 (4)
C9	0.0274 (5)	0.0291 (5)	0.0269 (5)	−0.0019 (4)	0.0164 (5)	−0.0024 (4)
S10	0.03026 (15)	0.03396 (16)	0.02658 (14)	0.00234 (11)	0.01480 (12)	0.00130 (11)
O11	0.0339 (5)	0.0434 (5)	0.0348 (5)	0.0094 (4)	0.0170 (4)	0.0007 (4)
O12	0.0481 (5)	0.0412 (5)	0.0368 (5)	−0.0024 (4)	0.0251 (4)	0.0049 (4)
C13	0.0250 (5)	0.0368 (6)	0.0252 (5)	0.0020 (5)	0.0128 (4)	−0.0014 (5)
C14	0.0310 (6)	0.0379 (6)	0.0312 (6)	0.0038 (5)	0.0195 (5)	0.0014 (5)
C15	0.0298 (6)	0.0368 (7)	0.0376 (6)	0.0029 (5)	0.0184 (5)	0.0011 (5)
C16	0.0307 (6)	0.0403 (7)	0.0361 (6)	0.0050 (5)	0.0144 (5)	−0.0054 (5)
C17	0.0389 (7)	0.0526 (8)	0.0371 (7)	0.0029 (6)	0.0243 (6)	−0.0091 (6)
C18	0.0338 (6)	0.0488 (8)	0.0344 (6)	−0.0013 (5)	0.0225 (5)	−0.0032 (6)
C19	0.0619 (10)	0.0455 (9)	0.0609 (10)	−0.0006 (8)	0.0329 (9)	−0.0153 (8)
N20	0.0362 (5)	0.0352 (6)	0.0396 (6)	0.0046 (4)	0.0262 (5)	0.0003 (4)
O21	0.0359 (5)	0.0473 (6)	0.0398 (5)	0.0108 (4)	0.0212 (4)	0.0028 (4)
O22	0.0555 (6)	0.0758 (8)	0.0550 (6)	0.0241 (6)	0.0431 (6)	0.0102 (6)
N23	0.0298 (5)	0.0347 (5)	0.0275 (5)	0.0063 (4)	0.0168 (4)	0.0003 (4)
C24	0.0280 (5)	0.0295 (6)	0.0237 (5)	0.0025 (4)	0.0143 (4)	−0.0010 (4)
C25	0.0305 (6)	0.0300 (6)	0.0270 (5)	0.0001 (5)	0.0154 (5)	−0.0012 (4)
N26	0.0302 (5)	0.0391 (6)	0.0256 (5)	−0.0045 (4)	0.0167 (4)	−0.0047 (4)
C27	0.0342 (6)	0.0523 (8)	0.0241 (5)	−0.0049 (6)	0.0148 (5)	−0.0061 (5)
C28	0.0301 (6)	0.0438 (7)	0.0293 (6)	−0.0065 (5)	0.0141 (5)	−0.0096 (5)
C29	0.0277 (6)	0.0364 (6)	0.0295 (5)	−0.0004 (5)	0.0151 (5)	−0.0022 (5)
C30	0.0423 (7)	0.0535 (9)	0.0483 (8)	−0.0141 (7)	0.0288 (7)	−0.0095 (7)
C31	0.0380 (7)	0.0584 (9)	0.0299 (6)	−0.0081 (6)	0.0204 (6)	−0.0000 (6)
C32	0.0321 (6)	0.0387 (7)	0.0314 (6)	−0.0006 (5)	0.0220 (5)	−0.0010 (5)
C33	0.0380 (6)	0.0351 (6)	0.0399 (6)	−0.0015 (5)	0.0254 (6)	0.0015 (5)
C34	0.0346 (7)	0.0518 (8)	0.0407 (7)	0.0045 (6)	0.0206 (6)	0.0081 (6)
C35	0.0359 (7)	0.0583 (9)	0.0445 (8)	−0.0103 (7)	0.0202 (6)	−0.0053 (7)
C36	0.0487 (8)	0.0378 (7)	0.0555 (9)	−0.0102 (6)	0.0320 (7)	−0.0051 (6)
C37	0.0420 (7)	0.0349 (6)	0.0422 (7)	0.0036 (5)	0.0280 (6)	0.0038 (5)

### Geometric parameters (Å, º)

**Table:**

N1—C9	1.3862 (15)	N23—C24	1.4595 (14)
N1—C2	1.3983 (14)	N23—H23	0.8659
N1—S10	1.6892 (10)	C24—C25	1.5258 (16)
C2—C3	1.3505 (17)	C24—C29	1.5305 (16)
C2—H2	0.9500	C24—H24	1.0000
C3—C4	1.4383 (15)	C25—N26	1.4616 (15)
C3—H3	0.9500	C25—H25A	0.9900
C4—C9	1.4042 (15)	C25—H25B	0.9900
C4—C5	1.4233 (16)	N26—C31	1.4682 (16)
C5—N23	1.3417 (15)	N26—C27	1.4706 (15)
C5—C6	1.4269 (15)	C27—C28	1.5166 (19)
C6—C7	1.4058 (17)	C27—H27A	0.9900
C6—N20	1.4335 (15)	C27—H27B	0.9900
C7—N8	1.3170 (17)	C28—C29	1.5279 (17)
C7—H7	0.9500	C28—H28A	0.9900
N8—C9	1.3436 (14)	C28—H28B	0.9900
S10—O12	1.4192 (10)	C29—C30	1.5275 (19)
S10—O11	1.4257 (10)	C29—H29	1.0000
S10—C13	1.7479 (13)	C30—H30A	0.9800
C13—C18	1.3879 (16)	C30—H30B	0.9800
C13—C14	1.3892 (18)	C30—H30C	0.9800
C14—C15	1.3794 (19)	C31—C32	1.5081 (18)
C14—H14	0.9500	C31—H31A	0.9900
C15—C16	1.3964 (18)	C31—H31B	0.9900
C15—H15	0.9500	C32—C37	1.3860 (19)
C16—C17	1.385 (2)	C32—C33	1.3902 (18)
C16—C19	1.506 (2)	C33—C34	1.384 (2)
C17—C18	1.387 (2)	C33—H33	0.9500
C17—H17	0.9500	C34—C35	1.381 (2)
C18—H18	0.9500	C34—H34	0.9500
C19—H19A	0.9800	C35—C36	1.378 (2)
C19—H19B	0.9800	C35—H35	0.9500
C19—H19C	0.9800	C36—C37	1.384 (2)
N20—O22	1.2234 (14)	C36—H36	0.9500
N20—O21	1.2427 (15)	C37—H37	0.9500
			
C9—N1—C2	108.32 (10)	N23—C24—C29	110.13 (9)
C9—N1—S10	127.01 (8)	C25—C24—C29	110.27 (9)
C2—N1—S10	124.35 (8)	N23—C24—H24	108.4
C3—C2—N1	109.26 (10)	C25—C24—H24	108.4
C3—C2—H2	125.4	C29—C24—H24	108.4
N1—C2—H2	125.4	N26—C25—C24	109.66 (10)
C2—C3—C4	107.90 (10)	N26—C25—H25A	109.7
C2—C3—H3	126.0	C24—C25—H25A	109.7
C4—C3—H3	126.0	N26—C25—H25B	109.7
C9—C4—C5	118.10 (10)	C24—C25—H25B	109.7
C9—C4—C3	106.68 (10)	H25A—C25—H25B	108.2
C5—C4—C3	135.16 (10)	C25—N26—C31	111.37 (10)
N23—C5—C4	123.54 (10)	C25—N26—C27	109.26 (10)
N23—C5—C6	123.33 (11)	C31—N26—C27	109.19 (10)
C4—C5—C6	113.13 (10)	N26—C27—C28	111.64 (10)
C7—C6—C5	121.94 (11)	N26—C27—H27A	109.3
C7—C6—N20	115.86 (10)	C28—C27—H27A	109.3
C5—C6—N20	122.15 (11)	N26—C27—H27B	109.3
N8—C7—C6	125.37 (11)	C28—C27—H27B	109.3
N8—C7—H7	117.3	H27A—C27—H27B	108.0
C6—C7—H7	117.3	C27—C28—C29	113.32 (11)
C7—N8—C9	112.50 (10)	C27—C28—H28A	108.9
N8—C9—N1	123.25 (10)	C29—C28—H28A	108.9
N8—C9—C4	128.92 (11)	C27—C28—H28B	108.9
N1—C9—C4	107.80 (10)	C29—C28—H28B	108.9
O12—S10—O11	120.63 (6)	H28A—C28—H28B	107.7
O12—S10—N1	107.84 (6)	C30—C29—C28	111.43 (11)
O11—S10—N1	103.40 (5)	C30—C29—C24	112.80 (11)
O12—S10—C13	110.18 (6)	C28—C29—C24	106.73 (10)
O11—S10—C13	109.71 (6)	C30—C29—H29	108.6
N1—S10—C13	103.51 (6)	C28—C29—H29	108.6
C18—C13—C14	121.34 (12)	C24—C29—H29	108.6
C18—C13—S10	119.65 (10)	C29—C30—H30A	109.5
C14—C13—S10	119.01 (9)	C29—C30—H30B	109.5
C15—C14—C13	119.13 (12)	H30A—C30—H30B	109.5
C15—C14—H14	120.4	C29—C30—H30C	109.5
C13—C14—H14	120.4	H30A—C30—H30C	109.5
C14—C15—C16	120.83 (13)	H30B—C30—H30C	109.5
C14—C15—H15	119.6	N26—C31—C32	114.09 (10)
C16—C15—H15	119.6	N26—C31—H31A	108.7
C17—C16—C15	118.77 (13)	C32—C31—H31A	108.7
C17—C16—C19	121.24 (13)	N26—C31—H31B	108.7
C15—C16—C19	119.98 (14)	C32—C31—H31B	108.7
C16—C17—C18	121.52 (12)	H31A—C31—H31B	107.6
C16—C17—H17	119.2	C37—C32—C33	118.27 (12)
C18—C17—H17	119.2	C37—C32—C31	120.14 (12)
C17—C18—C13	118.40 (13)	C33—C32—C31	121.51 (12)
C17—C18—H18	120.8	C34—C33—C32	120.95 (13)
C13—C18—H18	120.8	C34—C33—H33	119.5
C16—C19—H19A	109.5	C32—C33—H33	119.5
C16—C19—H19B	109.5	C35—C34—C33	120.09 (14)
H19A—C19—H19B	109.5	C35—C34—H34	120.0
C16—C19—H19C	109.5	C33—C34—H34	120.0
H19A—C19—H19C	109.5	C36—C35—C34	119.47 (14)
H19B—C19—H19C	109.5	C36—C35—H35	120.3
O22—N20—O21	121.26 (11)	C34—C35—H35	120.3
O22—N20—C6	119.17 (11)	C35—C36—C37	120.42 (14)
O21—N20—C6	119.57 (10)	C35—C36—H36	119.8
C5—N23—C24	126.22 (10)	C37—C36—H36	119.8
C5—N23—H23	113.6	C36—C37—C32	120.79 (13)
C24—N23—H23	117.8	C36—C37—H37	119.6
N23—C24—C25	111.27 (10)	C32—C37—H37	119.6
			
C9—N1—C2—C3	−1.84 (14)	C14—C15—C16—C17	−0.04 (19)
S10—N1—C2—C3	−175.67 (9)	C14—C15—C16—C19	−179.48 (13)
N1—C2—C3—C4	1.00 (14)	C15—C16—C17—C18	−0.3 (2)
C2—C3—C4—C9	0.19 (14)	C19—C16—C17—C18	179.08 (14)
C2—C3—C4—C5	177.15 (13)	C16—C17—C18—C13	0.7 (2)
C9—C4—C5—N23	−178.74 (11)	C14—C13—C18—C17	−0.62 (19)
C3—C4—C5—N23	4.6 (2)	S10—C13—C18—C17	178.80 (10)
C9—C4—C5—C6	1.73 (15)	C7—C6—N20—O22	−4.61 (18)
C3—C4—C5—C6	−174.97 (13)	C5—C6—N20—O22	172.83 (13)
N23—C5—C6—C7	178.66 (12)	C7—C6—N20—O21	176.09 (12)
C4—C5—C6—C7	−1.81 (17)	C5—C6—N20—O21	−6.46 (18)
N23—C5—C6—N20	1.37 (18)	C4—C5—N23—C24	19.43 (19)
C4—C5—C6—N20	−179.10 (11)	C6—C5—N23—C24	−161.08 (11)
C5—C6—C7—N8	0.7 (2)	C5—N23—C24—C25	−100.89 (13)
N20—C6—C7—N8	178.17 (12)	C5—N23—C24—C29	136.51 (12)
C6—C7—N8—C9	0.48 (19)	N23—C24—C25—N26	173.52 (9)
C7—N8—C9—N1	177.44 (11)	C29—C24—C25—N26	−63.96 (12)
C7—N8—C9—C4	−0.51 (19)	C24—C25—N26—C31	−176.61 (10)
C2—N1—C9—N8	−176.39 (11)	C24—C25—N26—C27	62.69 (12)
S10—N1—C9—N8	−2.77 (18)	C25—N26—C27—C28	−57.92 (14)
C2—N1—C9—C4	1.92 (13)	C31—N26—C27—C28	−179.95 (12)
S10—N1—C9—C4	175.54 (9)	N26—C27—C28—C29	54.63 (15)
C5—C4—C9—N8	−0.68 (19)	C27—C28—C29—C30	−176.25 (11)
C3—C4—C9—N8	176.89 (12)	C27—C28—C29—C24	−52.70 (14)
C5—C4—C9—N1	−178.87 (10)	N23—C24—C29—C30	−57.42 (14)
C3—C4—C9—N1	−1.30 (13)	C25—C24—C29—C30	179.40 (11)
C9—N1—S10—O12	52.95 (12)	N23—C24—C29—C28	179.88 (10)
C2—N1—S10—O12	−134.39 (11)	C25—C24—C29—C28	56.70 (13)
C9—N1—S10—O11	−178.24 (11)	C25—N26—C31—C32	62.29 (15)
C2—N1—S10—O11	−5.58 (12)	C27—N26—C31—C32	−176.96 (12)
C9—N1—S10—C13	−63.80 (12)	N26—C31—C32—C37	−140.70 (13)
C2—N1—S10—C13	108.86 (11)	N26—C31—C32—C33	42.81 (18)
O12—S10—C13—C18	−4.55 (12)	C37—C32—C33—C34	0.88 (19)
O11—S10—C13—C18	−139.64 (10)	C31—C32—C33—C34	177.43 (12)
N1—S10—C13—C18	110.54 (10)	C32—C33—C34—C35	−0.7 (2)
O12—S10—C13—C14	174.88 (9)	C33—C34—C35—C36	−0.2 (2)
O11—S10—C13—C14	39.80 (11)	C34—C35—C36—C37	0.8 (2)
N1—S10—C13—C14	−70.03 (10)	C35—C36—C37—C32	−0.6 (2)
C18—C13—C14—C15	0.25 (18)	C33—C32—C37—C36	−0.25 (19)
S10—C13—C14—C15	−179.18 (9)	C31—C32—C37—C36	−176.85 (12)
C13—C14—C15—C16	0.09 (19)		

### Hydrogen-bond geometry (Å, º)

**Table:**

*D*—H···*A*	*D*—H	H···*A*	*D*···*A*	*D*—H···*A*
N23—H23···O21	0.87	1.93	2.6274 (14)	137
